# Supplementary material for: Factors associated with neonatal near miss among neonates admitted to public hospitals in dire Dawa administration, Eastern Ethiopia: A case-control study
Source: PLoS One. 2022 Aug 29;17(8):e0273665. doi: 10.1371/journal.pone.0273665 (PMC9423664; doi:10.1371/journal.pone.0273665)
Supplement: S1 Questionnaire — (DOCX) [file pone.0273665.s002.docx]

## **Questionnaire**

**Title of the study**: Factors associated with a neonatal near-miss among neonates in public hospital of Dire Dawa city administration, Eastern Ethiopia.

***Interview and Record Review Identification Number***

| **S.No** | **Questions** | **Response** | **Skip** |
| --- | --- | --- | --- |
| **Part I: Identifications** | | | |
|  | Date form filled | / / |  |
|  | Maternity record number/Medical Reg. |  |  |
| **Part II: Socio-demographic and economic characteristics** | | | |
| 101 | Maternal age? | (in completed year) |  |
| 102 | Resident | 1. Urban 2. Rural |  |
| 103 | What is your (mother’s) educational status? | 1. No formal education 2. Primary (1-8) 3. Secondary (9-12) 4. College and above |  |
| 104 | What is the occupation of the mother? | 1. House wife 2. Merchant 3. Government employer 4. Non-governmental/private 5. Daily laborer 6. Others (specify) _________ |  |
| 105 | What is your (maternal) marital status? | 1. Single 2. Married 3. Widowed 4. Divorced | If not 2 skip to 108 |
| 107 | What is paternal educational status? | 1. No formal education 2. Primary (1-8) 3. Secondary (9-12) 4. College and above |  |
| 108 | What is your religion? | 1. Orthodox 2. Muslim 3. Protestant 4. Catholic 5. Others (specify) __________ |  |
| 109 | How much is your family average income per month? | (ETB) |  |
| **Part III: Maternal Factors related questions (Record review if it is available, if not interview the mother)** | | | |
| 201 | Pregnancy status | 1. Planned and wanted 2. Unplanned but wanted 3. Unplanned and unwanted |  |
| 202 | Number of pregnancies (gravidity)? | ____(in number) **If primi** | **208** |
| 203 | Number of birth orders (parity)? | (in number) | 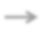 |
| 204 | Do you have pervious history of stillbirth? | 1. Yes 2. No |  |
| 205 | Do you have pervious history of abortion? | 1. Yes 2. No |  |
| 206 | The duration between the current birth and the preceding birth in months? | (in months) |  |
| 207 | Do you have pervious history of neonatal death? | 1. Yes 2. No | 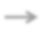 |
| 208 | Did you (the woman) received antenatal care for current pregnancy? | 1. Yes 2. No | **210** |
| 209 | If yes, number of ANC visits | **_________** |  |
| 210 | Women’s referred from other health facility | 1. Yes 2. No |  |
| **Part IV: Obstetric related questions (Record review/interview)** | | | |
| 301 | Labor status | 1. Spontaneous 2. Induced |  |
| 302 | Multiple births | 1. Yes 2. No |  |
| 303 | Duration of labor | ______ (in hours) |  |
| 304 | Developed any complication? | 1. Yes 2. No |  |
| 305 | Time of complication | 1. Occurred before admission 2. Occurred after admission |  |
| 306 | Dystocia | 1. Yes 2. No | **308** 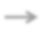 |
| 307 | If yes to question number 306, which one of these? | 1. Uterine pre-rupture 2. Prolonged labor 3. Foeto-pelvic disproportion |  |
| 308 | Mode of delivery | 1. Spontaneous vaginal delivery 2. Instrumental assisted delivery 3. Caesarean section |  |
| 309 | Hemorrhage | 1. Yes 2. No | **311** 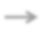 |
| 310 | If yes to the above question what was the cause? | 1. Placenta praevia 2. Placenta accreta/increta/percreta 3. Retro-placental haematoma 4. Other first trimester hemorrhage 5. Hemorrhage during delivery 6. Uterine rupture 7. Postpartum hemorrhage 8. Other obstetric hemorrhage (specify) ___________ |  |
| 311 | Premature rupture of membrane (PROM) | 1. Yes 2. No |  |
| 312 | Previous cesarean section | 1. Yes 2. No |  |
| 313 | Raptured uterus | 1. Yes 2. No |  |
| 314 | Hypertensive during pregnancy (HDP) | 1. Yes 2. No | **316** |
| 315 | If yes classifications of HDP | 1. Gestational hypertension 2. Mild preeclampsia 3. Severe preeclampsia 4. Eclampsia 5. Chronic hypertension | 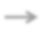 |
| 316 | Presence of anemia (Hgb <11g/dl) | 1. Yes 2. No |  |
| 317 | Maternal infection | 1. Yes 2. No | **319** |
| 318 | If yes to question number **317**, specify it | 1. Unspecified infection 2. Puerperal endometritis 3. Pyelonephritis 4. Septicaemia 5. Peritonitis 6. Syphilis 7. Other systemic infection | 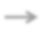 |
| 319 | Other pathologies | 1. Yes 2. No | **401** |
| 320 | If Yes | 1. HIV/AIDS 2. Tuberculosis 3. Malaria 4. DM 5. Embolic diseases 6. Heart disease 7. Sickle-cell disease 8. Other (specify): _________ | 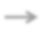 |
| **Part V: Newborn related** | | | |
| 401 | Presentation | 1. Cephalic 2. Breech 3. Transverse/face/brow 4. Other, specify__________ |  |
| 402 | Sex | 1. Male 2. Female |  |
| 403 | Birth trauma | 1. Yes 2. No |  |
| 404 | Non-reassuring fetal heart rate pattern | 1. Yes 2. No |  |

***Identification criteria for Neonatal near miss***

| **Part VI: Neonatal Near-Miss Criteria’s** | | | |
| --- | --- | --- | --- |
| **Pragmatic Markers** | | | |
| 501 | Gestational age | ________(in weeks) |  |
| 502 | Birth weight | ________(gm) |  |
| 503 | APGAR score at 5^th^ minute | ______ |  |
| **Management Severity Criteria’s** | | | |
| 504 | Use of mechanical ventilation | 1. Yes 2. No |  |
| 505 | Nasal continuous positive airway pressure (CPAP) | 1. Yes 2. No |  |
| 506 | Any intubation | 1. Yes 2. No |  |
| 507 | Use of parenteral antibiotic therapy | 1. Yes 2. No |  |
| 508 | Use of phototherapy in the first 24 hour | 1. Yes 2. No |  |
| 509 | Cardio pulmonary resuscitation | 1. Yes 2. No |  |
| 510 | Use of any vasoactive drug | 1. Yes 2. No |  |
| 511 | Use of anticonvulsants | 1. Yes 2. No |  |
| 512 | Use of surfactant | 1. Yes 2. No |  |
| 513 | Transfusion of blood derivatives | 1. Yes 2. No |  |
| 514 | Use of corticosteroid for treatment of  refractory hypoglycemia | 1. Yes 2. No |  |
| 515 | Any surgical procedure | 1. Yes 2. No |  |
| 516 | Use of antenatal steroid | 1. Yes 2. No |  |
| 517 | Parenteral nutrition | 1. Yes 2. No |  |
| 518 | Congenital malformation –ICD-10 | 1. Yes 2. No |  |
| 519 | Cord accident | 1. Yes 2. No |  |
| 520 | Admission to NICU | 1. Yes 2. No |  |
| 521 | **Is the newborn considered a near miss?** | 1. Yes 2. No |  |

***Thank you for your cooperation!***
