# Supplementary material for: Factors associated with neonatal near miss among neonates admitted to public hospitals in dire Dawa administration, Eastern Ethiopia: A case-control study
Source: PLoS One. 2022 Aug 29;17(8):e0273665. doi: 10.1371/journal.pone.0273665 (PMC9423664; doi:10.1371/journal.pone.0273665)
Supplement: S1 Ethics letter — (PDF) [file pone.0273665.s003.pdf]

ድሬዳዋ ዩኒቨርሲቲ
ምርምርና ማህበረሰብ አገልግሎት ም/ፕሬዝዳንት ጽ/ቤት

Dire Dawa University
Research & Community Services
V/President Office

☎ 025-112-77-80 ✉ 1362 ፋክስ 025-1-12-79-77 ድሬዳዋ ኢትዮጵያ
E-Mail @ddu.edu.et FAX Dire Dawa Ethiopia

Ref. ም/ማ/አ/ም/ፕ300/ 879/ 2013
ቁጥር
Date: ህዳር 2 /03 /2013

**Subject:** Letter of Confirmation for Ethical Clearance and Research Work

## To Whom It May Concern,

This is to certify that **Yitagesu Sintayehu** is Dire Dawa University Academic Staff and currently working on a research in title **"Factors Associated With Neonatal Near Miss Among Neonates In Public Hospital Of Dire Dawa City Administration, Eastern Ethiopia; A Case Control Study."**. The researcher has given Ethical Clearance from Dire Dawa University Research Ethics Review Committee to proceed the research process on **November 4-2020** . The RERC has given full considerations in relation to Ethical, Legal and Moral questions which may arise from the protocol.

Since the research work which s/he is doing holds a valuable importance to the local community, the University and Government, we kindly request your good office essential support for the information's required and data collection in the research process.

Dire Dawa University highly values and recognizes your good will, indispensible cooperation and support for the smooth processing and completion of the research work!!

Thanking in advance

With kind regards,

ሰለሞን ዘሪሁን (ዶ/ር)
Solomon Zerihun (PhD)
Research & Community
Service V/president

Federal Democratic Republic of Ethiopia
Dire-Dawa University
