# Supplementary material for: Factors associated with neonatal near miss among neonates admitted to public hospitals in dire Dawa administration, Eastern Ethiopia: A case-control study
Source: PLoS One. 2022 Aug 29;17(8):e0273665. doi: 10.1371/journal.pone.0273665 (PMC9423664; doi:10.1371/journal.pone.0273665)
Supplement: Attachment — Submitted filename: General comments.docx [file pone.0273665.s004.docx]

**General comments**

Dear authors on your scholarly work; you have brought an important study problem with good findings that have public health importance in the area of practice. Moreover, the manuscript has been written in good English. However, it still needs improvement for mainly grammar usage so that its readership becomes increased if published.

Some of the language use errors which the authors made consistently include.

**Eg.**

a. Card review……… instead of chart review
b. Bivariate and multivariate ….. instead of bivariable and multivariable

# Specific comments

1. **Background** of the abstract doesn’t clearly show the existing numerical burden of the problem in Dire Dawa town or other regional states of Ethiopia even. Generally, burden of neonatal near miss should be stated numerically followed by the objectives showing the research gap the authors would like to address.
2. The fourth sentence of the background that reads as ‘However, little studies were done in other areas, but they failed in identifying the proximate factors and not done in Dire Dawa Administration’ should be rewritten as: Though there were few prior studies in other regions, they failed in identifying the proximate factors. Besides, there has been no prior study in the study area.

# 3. Methods of abstract should include sampling technique, measurement of neonatal nearmiss and type of data collection tool (adapted or adopted)

# 4. Result of abstract: please include response rate in the first sentence of the result section.

# Introduction

# 5. Well written except the need for further synthesis of redundant concepts.

**Methods**

# 6. Data collection tools, procedures, and quality control: It would be more self explanatory and easily understandable if the authors showed pictorial presentation (flow chart) of the sampling procedure to reach a response rate of 100%. Please upload your data collection tools as additional file than annexing it in the manuscript. Kindly state the exact number of cases and controls rather than mentioning the routine statistics i.e. 5% of the sample size.

# 7. Ethical consideration: kindly have a separate upload of the ethical letter as an additional file. Moreover, what beneficent actions did the authors provide the mothers and the facilities in return for the interviews and chart reviews?

8. **Results**

How were you able to measure monthly income because you haven’t planned principal component analysis?

9. **Conclusion**

Well supported by the findings
